# Comparative biological activity of palbociclib and ribociclib in hormone receptor-positive breast cancer

**DOI:** 10.1038/s41598-024-67126-2

**Published:** 2024-07-11

**Authors:** Natàlia Lorman-Carbó, Olga Martínez-Sáez, Aranzazu Fernandez-Martinez, Patricia Galván, Nuria Chic, Isabel Garcia-Fructuoso, Adela Rodríguez, Raquel Gómez-Bravo, Francesco Schettini, Paula Blasco, Oleguer Castillo, Blanca González-Farré, Barbara Adamo, Maria Vidal, Montserrat Muñoz, Charles M. Perou, Marcos Malumbres, Joaquín Gavilá, Tomás Pascual, Aleix Prat, Fara Brasó-Maristany

**Affiliations:** 1 Translational Genomics and Targeted Therapies in Solid Tumors, August Pi I Sunyer Biomedical Research Institute (IDIBAPS), Carrer de Casanova, 143, 08036 Barcelona, Spain; 2 Medical Oncology Department, Hospital Clinic of Barcelona, Barcelona, Spain; 3 Lineberger Comprehensive Cancer Center, University of North Carolina, Chapel Hill, NC USA; 4 Department of Genetics, University of North Carolina, Chapel Hill, NC USA; 5 Division of Research, Peter MacCallum Cancer Centre, Melbourne, Australia; 6 Present Address: University of Barcelona, Barcelona, Spain; 7 Pathology Department, Hospital Clinic of Barcelona, Barcelona, Spain; 8 SOLTI Cooperative Group, Barcelona, Spain; 9 Institute of Oncology-Hospital Quirónsalud, Barcelona, Spain; 10 Cancer Cell Cycle Group, Vall d’Hebron Institute of Oncology (VHIO), Vall d’Hebron Barcelona Hospital Campus, Barcelona, Spain; 11 ICREA, Barcelona, Spain; 12 Department of Medical Oncology, Instituto Valenciano de Oncología, Valencia, Spain; 13 Reveal Genomics, S.L, Barcelona, Spain

**Keywords:** Breast cancer, Cell biology

## Abstract

This study examines the biological effects of palbociclib and ribociclib in hormone receptor-positive breast cancer, pivotal to the HARMONIA prospective phase III clinical trial. We explore the downstream impacts of these CDK4/6 inhibitors, focusing on cell lines and patient-derived tumor samples. We treated HR+ breast cancer cell lines (T47D, MCF7, and BT474) with palbociclib or ribociclib (100 nM or 500 nM), alone or combined with fulvestrant (1 nM), over periods of 24, 72, or 144 h. Our assessments included PAM50 gene expression, RB1 phosphorylation, Lamin-B1 protein levels, and senescence-associated β-galactosidase activity. We further analyzed PAM50 gene signatures from the CORALLEEN and NeoPalAna phase II trials. Both CDK4/6 inhibitors similarly inhibited proliferation across the cell lines. At 100 nM, both drugs partially reduced p-RB1, with further decreases at 500 nM over 144 h. Treatment led to reduced Lamin-B1 expression and increased senescence-associated β-galactosidase activity. Both drugs enhanced Luminal A and reduced Luminal B and proliferation signatures at both doses. However, the HER2-enriched signature significantly diminished only at the higher dose of 500 nM. Corresponding changes were observed in tumor samples from the CORALLEEN and NeoPalAna studies. At 2 weeks of treatment, both drugs significantly reduced the HER2-enriched signature, but at surgery, this reduction was consistent only with ribociclib. Our findings suggest that while both CDK4/6 inhibitors effectively modulate key biological pathways in HR+/HER2- breast cancer, nuances in their impact, particularly on the HER2-enriched signature, are dose-dependent, influenced by the addition of fulvestrant and warrant further investigation.

## Introduction

The addition of cyclin-dependent kinase 4/6 (CDK4/6) inhibitors to endocrine therapy in the treatment of patients with advanced hormone receptor-positive and HER2-negative (HR+/HER2-) breast cancer has significantly improved survival outcomes becoming the first-line standard of care therapy in this group of patients^1–11^. However, not all patients benefit to the same extent and efforts to identify biomarkers of sensitivity and resistance are ongoing.

HR+/HER2- breast cancer can be classified into four main molecular subtypes using gene expression profiling (PAM50) (i.e., Luminal A, Luminal B, HER2-enriched, and Basal-like)^12^. Of note, 5%–20% of HR+/HER2- tumors do not fall into the Luminal A or B subtypes but rather fall into the HER2-enriched phenotype^13,14^. Moreover, a higher proportion of the HER2-enriched subtype is detected in metastases compared to primary HR+/HER2- tumors, while the proportion of the Luminal A subtype is lower in metastases and the proportion of Luminal B and Basal-like subtypes is similar in metastases and primary tumors^15^.

The ability of the molecular subtypes to predict benefit from CDK4/6 inhibitors in breast cancer has been evaluated in samples from the PALOMA-2^16^, the NeoPalAna^17^, and the MONALEESA-2,-3, and -7^18^ studies. A retrospective analysis of the PALOMA-2 trial, which randomized patients with HR+/HER2‒ advanced breast cancer to letrozole +/− palbociclib, analyzed the molecular subtype of 455 tumors. Whilst Luminal A (50%) and Luminal B (30%) subtypes benefited from the addition of palbociclib to letrozole, the HER2-enriched (18.7%) and Basal-like (0.5%) subtypes were associated with worse progression-free survival (PFS) in both treatment arms compared to the Luminal A group^16^. In the NeoPalAna study, which evaluated the effects of palbociclib plus anastrazole in patients with primary breast cancer, PAM50 subtype was determined in 32 tumors at baseline. Of note, two tumors with non-luminal subtypes were identified, both of which were resistant to palbociclib^17^. Additionally, the SOLTI-1303 PATRICIA study of palbociclib and trastuzumab in HR+/HER2 + advanced breast cancer showed that the Luminal A and B subtypes benefited substantially from palbociclib, while the HER2-enriched group had very small absolute benefit^19^. In contrast, a retrospective pooled analysis of the MONALEESA-2, -3, and -7 pivotal trials with ribociclib and endocrine therapy evaluated the PAM50 subtype of 1,160 tumor samples. Except for Basal-like tumors (2.6%), all other intrinsic subtypes showed a consistent PFS and overall survival (OS) benefit from the combination of endocrine therapy and ribociclib over endocrine therapy alone, with the HER2-enriched subtype (12.7%) exhibiting the highest relative and absolute benefit^18,20^.

In retinoblastoma (RB1)-competent cells, CDK4/6 inhibitors trigger cell cycle arrest by reducing the phosphorylation of downstream RB1 tumor suppressor protein and can also induce cellular senescence. Palbociclib and ribociclib are CDK4/6 inhibitors of similar structure that selectively bind to the ATP-binding pocket of CDK4 (palbociclib IC50 = 9–11 nM, ribociclib IC50 = 10 nM) and CDK6 (palbociclib IC50 = 15 nM, ribociclib IC50 = 39 nM)^21,22^. One could argue that despite both being CDK4/6 inhibitors their slightly different chemical structures^23,24^, mechanisms of action^25^ and pharmacokinetics^24–26^ might lead to dissimilarities in efficacy. On the other hand, in clinical practice palbociclib is given at a lower dose than ribociclib (125 mg daily vs 600 mg daily, respectively)^27^, which could indicate a dose-dependent efficacy of CDK4/6 inhibitors in this biologically aggressive subtype. These differences may have relevant clinical implications as ribociclib, but not palbociclib, has shown clinical benefit in other clinical scenarios such as high-risk early breast cancer^28–31^.

Here, we assess the biological changes that occur upon CDK4/6 inhibition in breast cancer cell lines and clinical samples of patients who participated in two neoadjuvant phase II studies of ribociclib or palbociclib in combination with endocrine therapy.

## Results

### Phenotypic changes in breast *cancer* cell lines during CDK4/6 inhibition

The biological changes that occur during CDK4/6 inhibition were assessed in T47D (HR+/HER2-/HER2-enriched), MCF7 (HR+/HER2-/Luminal B), and BT474 (HR+/HER2+ /HER2-enriched) breast cancer cell lines. Despite being HER2+ , the inclusion of the BT474 cell line to our study could be of relevance since it belongs to the HER2-enriched subtype by PAM50, and palbociclib has been shown to be less efficient in HR+/HER2+/HER2-enriched breast cancer^19^. Moreover, it has been shown that, except for the amplification and RNA/protein overexpression of *ERBB2* in HER2+ tumors, very minor biological differences exist at DNA, RNA, and protein levels between HER2+/HER2-enriched and HER2-/HER2-enriched tumors^32^.

**Figure 1:**
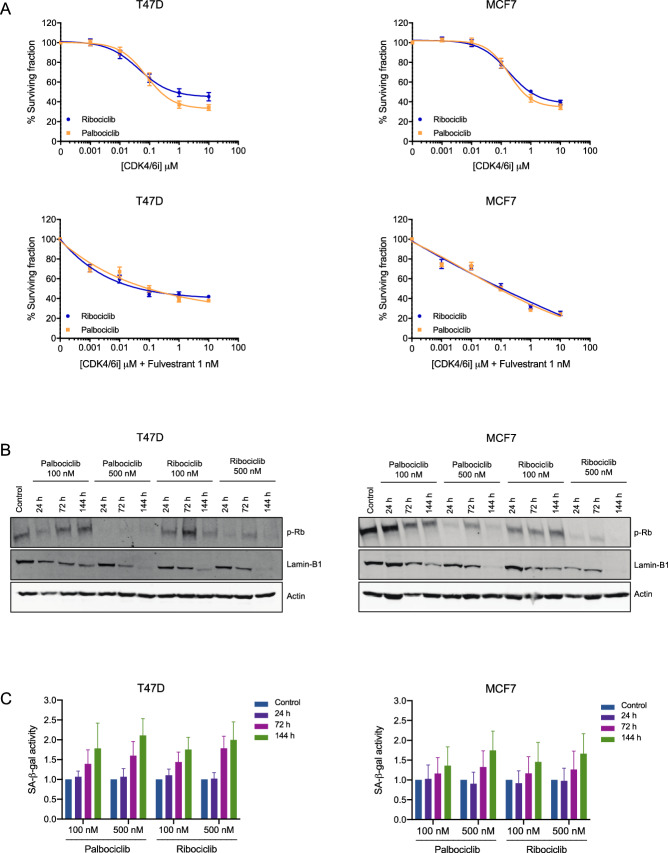
Biological changes during CDK4/6 inhibition in vitro. **(A)** T47D and MCF7 cells were treated with increasing doses of palbociclib or ribociclib +/− fulvestrant (1 nM) for 72 h. Shown are representative graphs of cell viability redouts determined by Hoechst 33342. Data was normalised to untreated cells and three independent experiments were performed. Mean values ± SEM are shown. **(B)** T47D and MCF7 cells were treated with palbociclib or ribociclib (100 or 500 nM) for 24, 72 or 144 h and expression of p-RB1 and Lamin-B1 was assessed by western blot. Actin was used as a loading control. **(C)** T47D and MCF7 cells were treated with palbociclib or ribociclib (100 or 500 nM) for 24, 72 or 144 h and SA-β-gal activity was determined by flow cytometry. Data was normalised to untreated cells and three independent experiments were performed. Mean values ± SEM are shown.

First, we analyzed the proliferation inhibitory effect of palbociclib and ribociclib +/− fulvestrant against all three cell lines and found that both CDK4/6 inhibitors had very similar effects (Fig. 1A, Supplementary Fig. 1A). In T47D and MCF7 cells, the combination of CDK4/6 inhibitors plus fulvestrant was superior than palbociclib or ribociclib alone, while in BT474 no significant differences were observed between the combination of CDK4/6 inhibitors with fulvestrant and palbociclib or ribociclib alone (Supplementary Fig. 2A).

RB1-competent T47D and MCF7 cell lines were treated with two different doses of palbociclib or ribociclib (i.e., 100 or 500 nM) for different periods of time (i.e., 24, 72, or 144 h) in order to assess changes in the phosphorylation of RB1 (p-RB1). In T47D and MCF7 cells treatment with 100 nM CDK4/6 inhibitors partially reduced p-RB1 with a further decrease in cells treated with 500 nM palbociclib or ribociclib regardless of treatment duration (Fig. 1B). Total *RB1* mRNA expression did not change after CDK4/6 inhibition (Supplementary Fig. 3). The cytotoxic effect of palbociclib and ribociclib was also assessed in the *RB1*-mutated MDA-MB-468 cell line to ensure that no off-target effects were given with the chosen doses (i.e., 100 or 500 nM) (Supplementary Fig. 2B).

Next, the effect on cellular senescence was assessed upon treatment with CDK4/6 inhibitors by determining protein expression levels of Lamin-B1, a structural component of the nucleus whose loss has been associated with senescence^33,34^. In T47D and MCF7 cells, CDK4/6 inhibitors decreased the expression of Lamin-B1 in a time and dose-dependent manner, with the lowest expression levels corresponding to those treated with 500 nM palbociclib or ribociclib for 144 h (Fig. 1B). Additionally, we assessed the senescence-associated β-galactosidase (SA-β-gal) activity. Both palbociclib and ribociclib (100 or 500 nM) increased β-galactosidase staining in T47D and MCF7 cell lines after 72 or 144 h treatments compared to non-treated controls indicating induction of senescence^35^ (Fig. 1C). Although no significant differences were observed between treatment conditions, a dose- and time-related tendency was noticeable, with the highest β-galactosidase staining levels corresponding to cells treated with 500 nM CDK4/6 inhibitors for 144 h. Similar changes were observed in cells treated with palbociclib or ribociclib for all treatment conditions (Fig. 1C).

As for the RB1-competent BT474 cell line, a reduction of p-RB1 was also observed upon 24 or 72 h treatments with CDK4/6 inhibitors (100 or 500 nM), although p-RB1 levels were reestablished 144 h from treatment (Supplementary Fig. 1B). Lower levels of β-galactosidase activity were detected generally and the highest activity was detected after 72 h treatments with either CDK4/6 inhibitor, followed by a decrease in cellular senescence 144 h from treatments (Supplementary Fig. 1C). These observations indicate that the BT474 cell line may harbor an intrinsic resistance to CDK4/6 inhibition +/− fulvestrant compared to the T47D and MCF7 cell lines.

### Effects of CDK4/6 inhibition on gene expression in breast *cancer* cell lines

**Figure 2:**
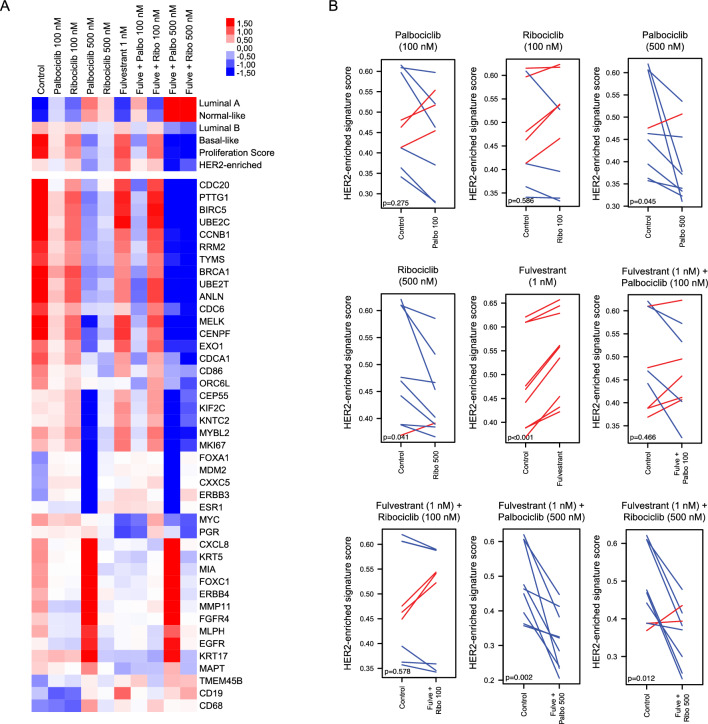
Changes in the HER2-enriched signature upon treatment with CDK4/6 inhibitors +/− fulvestrant in vitro. **(A)** Heatmap of a multiclass SAM representing the PAM50 molecular subtypes, proliferation score and genes that are differentially expressed (FDR < 5%) in T47D, MCF7, and BT474 cells treated with CDK4/6 inhibitors (100 or 500 nM) +/− fulvestrant (1 nM). Three independent mRNA extractions per cell line were performed. **(B)** Paired samples t-test analyses showing changes in the HER2-enriched signature following treatment of T47D, MCF7, and BT474 cells with CDK4/6 inhibitors (100 or 500 nM) +/− fulvestrant (1 nM). Three independent mRNA extractions and gene expression analyses were performed for each cell line.

Gene expression profiling was performed in untreated cells and upon treatment in order to identify changes in the PAM50 biology induced by CDK4/6 inhibitors in T47D, MCF7, and BT474 cell lines treated with different doses of palbociclib and ribociclib (100 or 500 nM) +/− fulvestrant (1 nM). The expression of the 50 genes of the PAM50 intrinsic subtype predictor and 6 signatures (Basal-like, HER2-enriched, Luminal A, Luminal B, Normal-like, and the 11-gene proliferation score) were explored at both treatment conditions. Paired t-tests and multiclass SAM showed that both CDK4/6 inhibitors (100 or 500 nM) +/− fulvestrant (1 nM) significantly increased (FDR < 5%) the Luminal A and Normal-like signatures and significantly decreased (FDR < 5%) the Basal-like and proliferation signatures (Fig. 2A, Supplementary Fig. 4). Interestingly, the HER2-enriched signature was only significantly reduced when the CDK4/6 inhibitors were given at 500 nM either alone (palbociclib p = 0.045, ribociclib p = 0.041) or in combination with fulvestrant (palbociclib p = 0.002, ribociclib p = 0.012), while no significant changes were observed with 100 nM CDK4/6 inhibitor monotherapy (palbociclib p = 0.275, ribociclib p = 0.596) or in combination with fulvestrant (palbociclib p = 0.466, ribociclib p = 0.613) (Fig. 2A,B). Treatment with fulvestrant alone significantly increased the HER2-enriched signature (p < 0.001) (Fig. 2A,B).

Next, we assessed individual gene expression across treatments using multiclass SAM. Forty-three (64.2%) genes were differentially expressed across treatment groups (FDR < 5%). Notably, both inhibitors, especially at 500 nM, led to a lower expression of proliferative genes (e.g.: *CDC20*, *UBE2C*, *KNTC2*, *MKI67*, *BIRC5*, *CDCA1*, *PTTG1*, *CEP55*, *TYMS*, and *RRM2*). Interestingly, in lower doses of CDK4/6 inhibitors (100 nM) the combination of fulvestrant and palbociclib had a stronger inhibitory effect over cell proliferation than the combination of fulvestrant and ribociclib. Additionally, we performed a paired SAM analysis to check the differences between 500 nM of palbociclib vs 500 nM ribociclib with or without fulvestrant. A proportion of 19.4% and 25.3% of genes were differentially expressed after treatment with 500 nM of palbociclib vs 500 nM ribociclib with or without fulvestrant, respectively (Supplementary Table 1). Interestingly, a higher expression of the HER2-enriched genes *FGFR4* and *TMEM45B* was observed in cells treated with 500 nM palbociclib compared to those treated with 500 nM ribociclib with and without fulvestrant (Fig. 2A).

### Early in vivo biological changes during CDK4/6 inhibitor in tumor samples from CORALLEEN and NeoPalAna phase II studies

**Figure 3:**
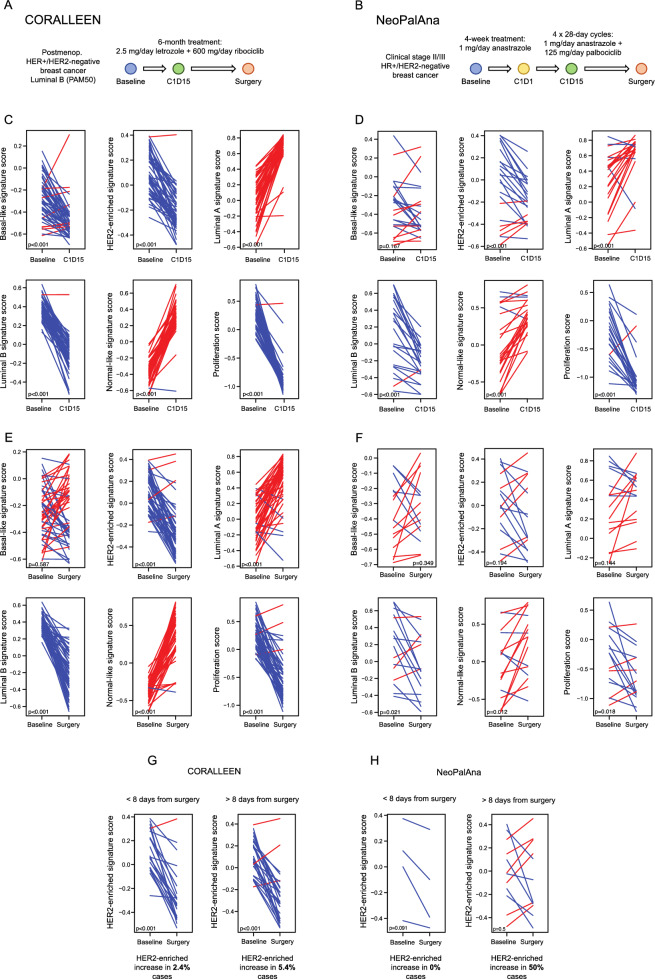
Changes in the PAM50 signatures in the CORALLEEN and NeoPalAna studies. Schematic summaries of the samples analyzed from **(A)** the CORALLEEN trial design and **(B)** the NeoPalAna trial design. **(C)** Paired samples t-test analyses showing changes in the PAM50 signatures at cycle 1 day 15 (C1D15) in tumor samples from CORALLEEN and **(D)** NeoPalAna phase II studies. **(E)** Changes in the PAM50 signatures at surgery in tumor samples from CORALLEEN and **(F)** NeoPalAna. **(G)** Changes in the HER2-enriched signature in tumor samples from CORALLEEN and **(H)** NeoPalAna from patients who underwent surgery ≤ 8 or > 8 days from the last dose of CDK4/6 inhibitors + endocrine therapy.

To identify molecular changes induced by CDK4/6 inhibitors, we performed gene expression analyses in baseline, day 15, and surgery tumor samples of patients treated with ribociclib plus letrozole in the CORALLEEN trial (Fig. 3A) as well as in baseline, day 15, and surgery samples of patients treated with palbociclib plus anastrazole in the NeoPalAna trial (Fig. 3B).

First, we assessed early changes in 49 paired baseline and day 15 tumor samples from the CORALLEEN trial (Fig. 3C) and 23 paired baseline and day 15 tumor samples from the NeoPalAna trial (Fig. 3D). Treatment with ribociclib and endocrine therapy led to a significant increase in Luminal A (p < 0.001) and Normal-like (p < 0.001) signatures and a significant decrease in Basal-like (p < 0.001), HER2-enriched (p < 0.001), Luminal B (p < 0.001) and proliferation (p < 0.001) signatures (Fig. 3C). Similarly, treatment with palbociclib plus endocrine therapy led to a significant increase in Luminal A (p < 0.001) and Normal-like (p < 0.001) signatures and a significant decrease in HER2-enriched (p < 0.001), Luminal B (p < 0.001) and proliferation (p < 0.001) signatures (Fig. 3D).

### Biological changes after CDK4/6 inhibitor in tumor samples from CORALLEEN and NeoPalAna phase II studies

Next, we assessed changes in 49 paired baseline and surgery tumor samples from the CORALLEEN (Fig. 3E) and 16 paired baseline and surgery tumor samples from the NeoPalAna (Fig. 3F).

Treatment with ribociclib and endocrine therapy led to a significant increase in Luminal A (p < 0.001) and Normal-like (p < 0.001) signatures and a significant decrease in HER2-enriched (p < 0.001), Luminal B (p < 0.001), and proliferation (p < 0.001) signatures (Fig. 3E). Treatment with palbociclib plus endocrine therapy led to a significant increase in the Normal-like (p = 0.012) signature and a significant decrease in the Luminal B (p = 0.021) and proliferation signature (p = 0.018) (Fig. 3F). Importantly, the HER2-enriched signature did not decrease in surgical samples of patients treated with palbociclib (p = 0.194), although a difference in sample size could explain this result (Figs. 3F).

In CORALLEEN, the median number of days between the last dose of ribociclib and surgery was 13.1 days (range: 1–78)^36^, whereas in NeoPalAna the median number of days between the last dose of palbociclib and surgery was 29 days (range: 8–49), except for 8 patients who received additional 10–12 days of palbociclib immediately before surgery^17^. In patients from CORALLEEN, the HER2-enriched signature was significantly decreased in patients who underwent surgery at 8 days from the last dose of ribociclib or before (p < 0.001), as well as in those who underwent surgery after > 8 days from the last dose of ribociclib (p < 0.001) (Fig. 3G). In 4 patients from NeoPalAna who underwent surgery at 8 days from the last dose of palbociclib or before, a tendency of reduction in the HER2-enriched signature was also observed. However, in patients who underwent surgery after > 8 days from the last dose of palbociclib, the HER2-enriched signature increased in 50% of the cases (Fig. 3H).

## Discussion

In the last few years, three CDK4/6 inhibitors (i.e., palbociclib, ribociclib, abemaciclib) have been approved for the treatment of patients with metastatic HR+/HER2- breast cancer in combination with endocrine therapy^1–11^. While the three inhibitors are theoretically considered to provide a similar class effect, they have some chemical and pharmacological differences and are given at different doses^22,25^. Currently, no specific biomarkers are used to select the first-line CDK4/6 inhibitor^37^. On one side, the first-line trials using the CDK4/6 inhibitors ribociclib and palbociclib, despite demonstrating identical primary endpoint PFS results, have recently reported different OS results, with palbociclib not showing an OS benefit^38^. It is unknown if this is due to differences in the type of inhibitor, trial, patient population, or other features.

On the other side, it has been demonstrated that the PAM50 molecular subtypes are prognostic in patients treated with CDK4/6 inhibitors^16,18,19^ and accumulated evidence suggests that the combination of endocrine therapy with palbociclib might be less effective than combination with ribociclib in patients with advanced HR+/HER2- and HER2-enriched breast cancer. Indeed, retrospective analyses on samples of the MONALEESA-2,-3, and -7^18^ trials showed that patients that harbored HER2-enriched tumors exhibited a PFS and OS benefit from the combination of endocrine therapy and ribociclib, whereas those treated with endocrine therapy and palbociclib in the PALOMA-2^16^ did not benefit from the combination, even though the retrospective analysis of PALOMA-2 was not powered to study the effect across PAM50 subtypes. Nevertheless, this hypothesis has not been formally tested head-to-head, and the SOLTI-2101 HARMONIA^39^ prospective phase III trial (NCT05207709) is currently evaluating if the combination of ribociclib with endocrine therapy is superior to the combination with palbociclib in prolonging PFS in this particular subset of patients.

In order to better understand the molecular effects of palbociclib and ribociclib, we analyzed both cell lines and clinical samples treated with CDK4/6 inhibitors. Our main observations in breast cancer cell lines were that palbociclib and ribociclib had identical dose-dependent proliferation inhibition and that in CDK4/6 inhibitor-sensitive cell lines, both inhibitors reduced the levels of p-RB1 (marker of cell cycle inhibition^21^) and Lamin-B1 (marker of senescence^40–42^) in a similar manner, where treatment duration played a part but changes mostly relied on the administered doses.

Importantly, gene expression analyses revealed that palbociclib and ribociclib significantly increased the Luminal A and Normal-like signatures and decreased the Luminal B, Basal-like, and proliferation signatures with both doses. However, the HER2-enriched signature was only significantly reduced in cells treated with 500 nM of CDK4/6 inhibitors +/− fulvestrant. Interestingly, treatment with fulvestrant alone significantly increased the HER2-enriched signature, but the addition of 500 nM palbociclib or ribociclib was still capable of significantly decreasing its levels. Assessment of individual gene expression suggested that in lower doses palbociclib might be more potent CDK4/6 inhibitor than ribociclib, and that co-treatment with fulvestrant further enhances these changes in gene expression.

In patient tumor samples from the CORALLEEN and NeoPalAna phase II studies a similar change in PAM50 biology was observed with both drugs namely an increase in Luminal A and Normal-like signatures and a decrease in Luminal B and proliferation signatures after 2 weeks of treatment and at surgery. At 2 weeks of treatment the HER2-enriched signature was significantly decreased in both studies. However, the decrease in the HER2-enriched signature was only observed in surgical samples of patients treated with ribociclib, but not palbociclib. Interestingly, in patients from NeoPalAna who underwent surgery at 8 days from the last dose of palbociclib or before, a reduction of the HER2-enriched signature was observed, although it was not of statistical significance possibly due to sample size. This result is consistent with the results of the NeoPalAna trial, where a Ki67 rebound at surgery following palbociclib was observed in patients where palbociclib treatment was finalized > 8 days before surgery, while this washout was suppressed if patients received a cycle 5 of palbociclib^17^. If palbociclib was given until surgery, the effect could be as good as the effect of ribociclib. However, sample size in NeoPalAna was much smaller compared to CORALLEEN and this represents a limitation on the interpretation of the results.

Our study acknowledges several other limitations. Firstly, our analysis was limited to early-stage breast cancer tumor samples, as obtaining paired biopsies in a metastatic setting is challenging. This may restrict the applicability of our findings to more advanced disease stages. Secondly, while most of these samples were not HER2-enriched, we attempted to mitigate this by analyzing each PAM50 intrinsic subtype score as a continuous variable, since these scores are strictly related to the biological information provided by the PAM50 genes characterizing each breast cancer intrinsic subtype^43^. Nonetheless, we acknowledge this may not fully capture the complexities of HER2-enriched biology. Thirdly, there is an acknowledged gap in our understanding of the actual concentration of palbociclib and ribociclib that reaches the tumor in patients, which may differ from the prescribed doses and preclinical models, adding a layer of uncertainty to the direct translatability of our results to clinical practice. Fourthly, the specificity of the CORALLEEN trial to patients with PAM50 Luminal B disease narrows the breadth of our findings, potentially limiting their generalizability to other breast cancer subtypes. Lastly, our study did not include abemaciclib, which has been proposed to target additional CDKs in addition to CDK4/6^44,45^. These limitations highlight the need for further research in order to fully understand the implications of CDK4/6 inhibitors in varying contexts of breast cancer treatment.

In conclusion, our results show that biological responses to palbociclib and ribociclib are primarily dose-dependent and influenced by the addition of fulvestrant. Our findings suggest that while both CDK4/6 inhibitors effectively modulate key biological pathways in HR+/HER2- breast cancer, nuances in their impact, particularly on the HER2-enriched signature, warrant further investigation. The ongoing SOLTI-2101 HARMONIA trial^39^ will ultimately test which CDK4/6 inhibitor is best for continued response and survival benefit in patients with HER2-enriched breast cancer.

## Methods

### Cell lines and drugs

MCF7, T47D, BT474, and MDA-MB-468 cell lines were obtained from the American Type Culture Collection (ATCC, Manassas, VA, USA) and cultured in Dulbecco’s Modified Eagle Medium (DMEM)/nutrient mixture F-12 supplemented with 10% v/v heat-inactivated fetal bovine serum (FBS) (Gibco; Thermo Fisher Scientific Inc., Waltham, MA, USA), 1% GlutaMAX (Gibco; Thermo Fisher Scientific Inc.), and 1% Penicillin/Streptomycin (Sigma-Aldrich, Saint Louis, MO, USA) in a 37 °C, 5% CO_2_ humidified incubator. Cells were detached from flasks by incubation with 0.25% Trypsin–EDTA (1X) (Gibco; Thermo Fisher Scientific Inc.). Palbociclib, ribociclib, and fulvestrant were purchased from Selleckchem (Houston, TX, USA).

### Clinical samples

The SOLTI-1402 CORALLEEN phase II study (NCT03248427) randomized 106 postmenopausal women with stage I–IIIA HR+/HER2- breast cancer and Luminal B by PAM50 with histologically confirmed, operable primary tumour size of at least 2 cm in diameter as measured by magnetic resonance imaging (MRI). Patients were randomly assigned (1:1) to receive either six 28-days cycles of ribociclib (oral 600 mg once daily for 3 weeks on, 1 week off) plus daily letrozole (oral 2.5 mg/day) or four cycles of doxorubicin (intravenous 60 mg/m^2^) and cyclophosphamide (intravenous 600 mg/m^2^) every 21 days followed by weekly paclitaxel (intravenous 80 mg/m^2^) for 12 weeks^36^. Here, we analyzed formalin-fixed, paraffin-embedded (FFPE) tumor samples of the ribociclib plus letrozole arm, including 49 paired baseline versus cycle 1 day 15 (C1D15) samples and 49 paired baseline versus surgery samples.

Additionally, gene expression data of the NeoPalAna phase II trial (NCT01723774), which treated 50 patients with HR+/HER2- early breast cancer with anastrazole (1 mg daily) for 4 weeks, followed by four 28-day cycles of palbociclib (125 mg daily) plus anastrazole (1 mg daily)^17^, was downloaded from the Gene Expression Omnibus (GSE93204). We analyzed 23 paired baseline versus C1D15 samples and 16 paired baseline versus surgery samples.

### Ethics approval and consent to participate

This study was approved by the Ethics Committee at Hospital Clinic of Barcelona (HCB.2022.0086) and all methods were carried out in accordance with relevant guidelines and regulations. This study involves the use of tissue samples of patients that have received treatment with CDK4/6 inhibitors within the context of the CORALLEEN trial. These samples are stored in the biorepository of the Translational genomics and targeted therapies in solid tumors group at IDIBAPS as long as patients sign the specific informed consent of the collection.

### RNA extraction

RNA of tumor samples from the CORALLEEN study was extracted using the High Pure FFPET RNA isolation kit (Roche, Indianapolis, IN, USA) following manufacturer’s protocol. At least 1–5 10 μm FFPE slides were used for each tumor specimen and macrodissection was performed to avoid contamination with normal breast tissue if needed. MCF7, T47D, and BT474 cells were seeded in a 6-well plate at 150,000 cells per well and after overnight incubation medium was replaced with two different dose levels of palbociclib or ribociclib (i.e., 100 nM or 500 nM) +/− fulvestrant (1 nM) for 72 h (h). mRNA was extracted using QIAGEN’s RNeasy extraction kit (QIAGEN, Hilden, Germany) following manufacturer’s instructions.

### Gene expression analysis

The nCounter platform (NanoString Technologies, Seattle, WA, USA) analyzed RNA samples from tumor samples and cell lines. A minimum of 100 ng of total RNA was used to measure the expression of 50 genes of the PAM50 intrinsic subtype predictor assay and 5 housekeeping genes (*ACTB, MRPL19, PSMC4, RPLP0* and *SF3A1*). Expression counts were then normalized and the PAM50 signature scores (Basal-like, HER2-enriched, Luminal A and B, Normal-like) and the proliferation signature score were calculated using customized R scripts^43^. PAM50 molecular subtypes were calculated in the publicly available gene expression data from the NeoPalAna including 23 baseline samples, 23 week-2 samples and 16 surgery samples.

### In vitro cell growth assay

MCF7, T47D, BT474, and MDA-MB-468 cells were seeded in triplicate at 5,000 cells per well in 96-well plates. Following overnight incubation, cells were treated with five 1:10 serial dilutions of palbociclib or ribociclib starting at 10 µM. Cell viability was assessed after 72 h with Hoechst 33342 staining solution (Invitrogen, Thermo Fisher Scientific Inc.) and quantified using SynergyHT microplate reader and Windows based Gen5 software.

### Western blotting

MCF7, T47D, and BT474 cells were seeded in 6-well plates at 150,000 cells per well and after overnight incubation medium was replaced with palbociclib or ribociclib (100 or 500 nM). After 24, 72, or 144 h, cell lysates were obtained using radioimmunoprecipitation (RIPA) lysis and extraction buffer (Thermo Fisher Scientific Inc.) supplemented with protease inhibitors: 5 mM sodium fluoride, 1 mg/ml aprotinin, 1 mM phenylmethylsulfonyl fluoride, 1 mM sodium orthovanadate, 1 mM benzamidine, 1 mM leupeptin, and 1 mM dithiothreitol. Total protein extracts were quantified using the DC Protein Assay (BioRad Laboratories, Hercules, CA, USA) and 50 μg of proteins were separated in reducing conditions (2.5% β-mercaptoethanol) by SDS–PAGE and transferred to nitrocellulose membranes (BioRad Laboratories) for further processing, following standard western blotting procedures. The primary antibodies used in this study were phospho-RB1 (Ser807/811) (D20B12) and Lamin-B1 (D9V6H) from Cell Signaling Technologies (Danvers, MA, USA) and anti-actin (A2066) from Sigma-Aldrich. The secondary fluorescent antibody used was the IRDye 800CW Donkey anti-Rabbit IgG (LI-COR Biosciences, Lincoln, NE, USA). Fluorescent signal was acquired by the Odyssey Imaging System (LI-COR Biosciences).

### Senescence-associated β-galactosidase activity

MCF7, T47D, and BT474 cells were seeded in 24-well plates at 35,000 cells per well and after overnight incubation medium was replaced with palbociclib or ribociclib (100 or 500 nM). Following 24, 72, or 144 h treatments, senescence dye from the Senescence Assay Kit (Abcam, Cambridge, UK) was added to wells. Cells were incubated for 1–2 h in a 37 °C, 5% CO_2_ humidified incubator and the mean fluorescence was analysed by flow cytometry for the detection of β-galactosidase activity. Propidium iodide (Invitrogen, Thermo Fisher Scientific Inc.) was used as a viability marker. Untreated controls were added, as well as unstained controls for the evaluation of potential auto-fluorescence.

### Statistical analysis

Changes in gene expression and PAM50 signatures upon CDK4/6 inhibition were determined by paired t-tests and paired and multiclass significant analysis of microarray (SAM) with a false discovery rate (FDR) < 5%. These analyses were performed using R software. All statistical tests were two sided and the statistical significance level was set to p < 0.05. For in vitro cell growth, determination of half maximal inhibitory concentrations (IC50s), and senescence assays, GraphPad Prism was used for statistics.

## Supplementary Information

Supplementary Information.

## Data Availability

Investigators interested in data access and collaboration should contact the corresponding author. Access can be obtained for academic use only under a data transfer agreement and upon Ethics Committee approval.
