# Supplementary material for: Comparative biological activity of palbociclib and ribociclib in hormone receptor-positive breast cancer
Source: Sci Rep. 2024 Jul 11;14:16030. doi: 10.1038/s41598-024-67126-2 (PMC11239654; doi:10.1038/s41598-024-67126-2)
Supplement: Supplementary file 1 — Supplementary Information. [file 41598_2024_67126_MOESM1_ESM.pdf]

Comparative Biological Activity of Palbociclib and Ribociclib in Hormone Receptor-Positive Breast Cancer

Natàlia Lorman-Carbó[1], Olga Martínez-Sáez[1,2], Aranzazu Fernandez-Martinez[3,4], Patricia Galván[1], Nuria Chic[1,5], Isabel Garcia-Fructuoso[1,2], Adela Rodríguez[1,2], Raquel Gómez-Bravo[1,2], Francesco Schettini[1,2,6], Paula Blasco[1], Oleguer Castillo[1], Blanca González-Farré[1,7], Barbara Adamo[1,2], Maria Vidal[1,2,8,9], Montserrat Muñoz[1,2,8], Charles M. Perou[3,4], Marcos Malumbres[10-11], Joaquín Gavilá[8,12], Tomás Pascual[1,2,8], Aleix Prat[1,2,6,9,13], Fara Brasó-Maristany[1,13,*]

1. Translational Genomics and Targeted Therapies in Solid Tumors, August Pi i Sunyer Biomedical Research Institute (IDIBAPS).
2. Medical Oncology Department, Hospital Clinic of Barcelona, Barcelona, Spain.
3. Lineberger Comprehensive Cancer Center, University of North Carolina, Chapel Hill, NC, USA.
4. Department of Genetics, University of North Carolina, Chapel Hill, NC, USA.
5. Division of Research, Peter MacCallum Cancer Centre, Melbourne, Australia.
6. University of Barcelona, Barcelona, Spain.
7. Pathology Department, Hospital Clinic of Barcelona, Barcelona, Spain.
8. SOLTI cooperative group, Barcelona, Spain.
9. Institute of Oncology-Hospital Quirónsalud, Barcelona, Spain.
10. Cancer Cell Cycle Group, Vall d'Hebron Institute of Oncology (VHIO), Vall d'Hebron Barcelona Hospital Campus, Barcelona, Spain.
11. ICREA, Barcelona.
12. Department of Medical Oncology, Instituto Valenciano de Oncología, Valencia, Spain.
13. Reveal Genomics, S.L., Barcelona, Spain.

*, Corresponding author: Dr. Fara Brasó-Maristany, Translational Genomic and Targeted Therapies in Solid Tumors, August Pi i Sunyer Biomedical Research Institute (IDIBAPS), Carrer de Casanova, 143, 08036, Barcelona, Spain. Email: fbraso@recerca.clinic.cat

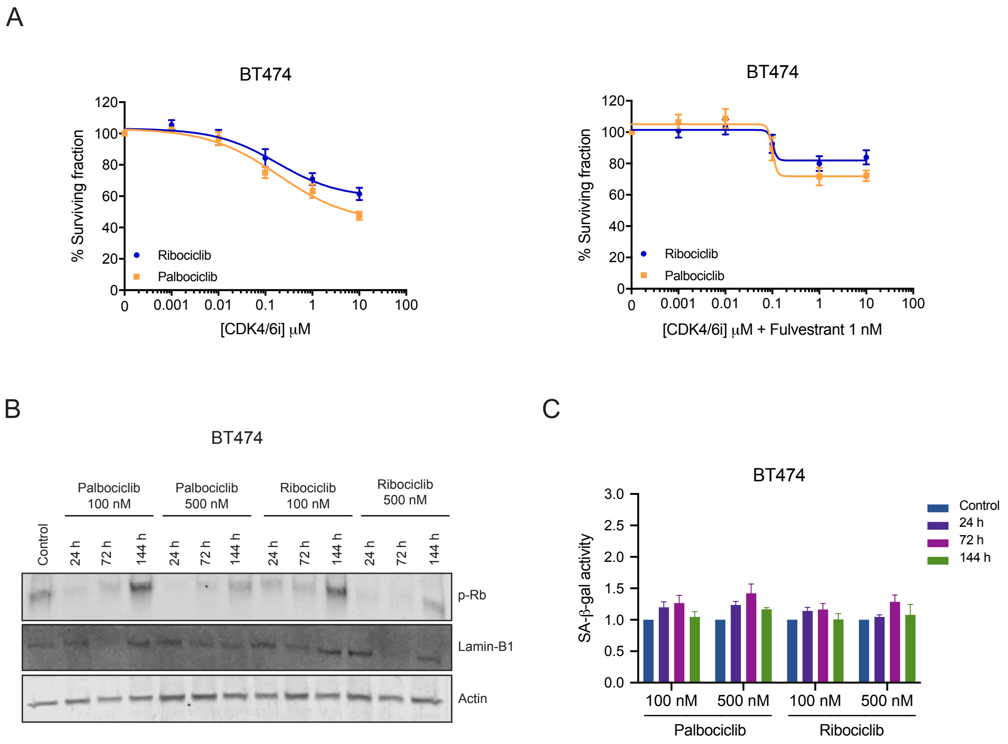

Supplementary Figure 1. Biological changes during CDK4/6 inhibition in vitro. (A) BT474 cells were treated with increasing doses of palbociclib or ribociclib +/- fulvestrant (1 nM) for 72 h. Shown are representative graphs of cell viability redouts determined by Hoechst 33342. Data was normalised to untreated cells and three independent experiments were performed. Mean values ± SEM are shown. **(B)** BT474 cells were treated with palbociclib or ribociclib (100 or 500 nM) for 24, 72 or 144 h and expression of p-RB1 and Lamin-B1 was assessed by western blot. Actin was used as a loading control. **(C)** BT474 cells were treated with palbociclib or ribociclib (100 or 500 nM) for 24, 72 or 144 h and SA-β-gal activity was determined by flow cytometry. Data was normalised to untreated cells and three independent experiments were performed. Mean values ± SEM are shown.

A

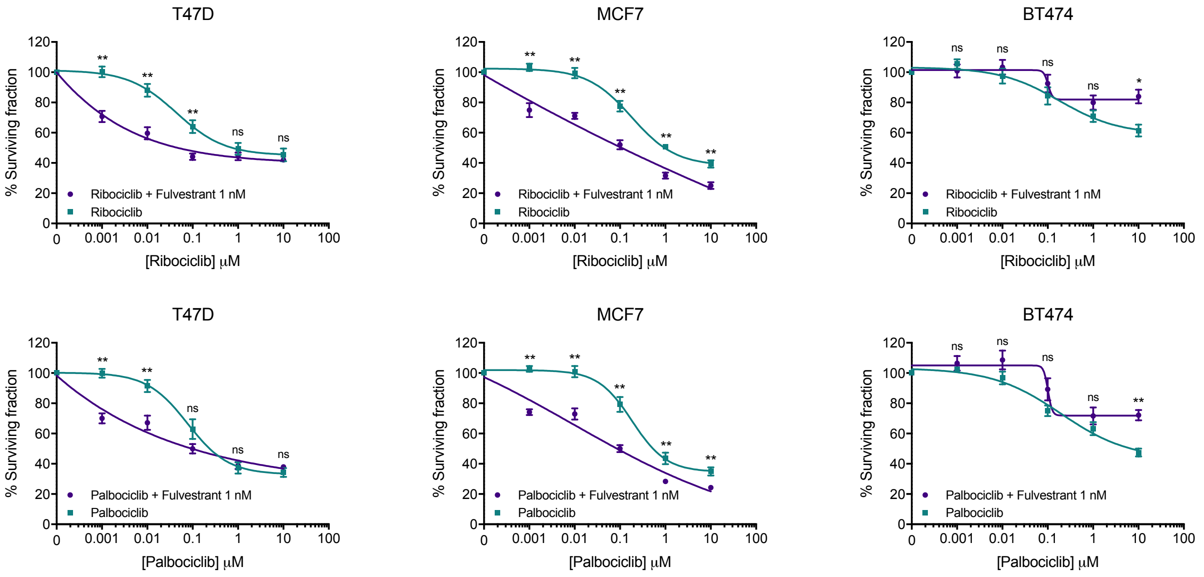

B

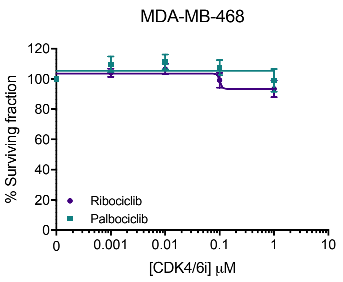

Supplementary Figure 2. Effects of CDK4/6 inhibition +/- fulvestrant in breast cancer cell lines. (A) MCF7, T47D, and BT474 cells were treated with increasing doses of palbociclib or ribociclib +/- fulvestrant (1 nM) for 72 h. Shown are representative graphs of cell viability redouts determined by Hoechst 33342. Data was normalised to untreated cells and three independent experiments were performed. Mean values ± SEM are shown. ns p>0.05, * p<0.05, ** p<0.001. **(B)** MDA-MB-468 cells were treated with increasing doses of palbociclib or ribociclib for 72 h. Shown are representative graphs of cell viability redouts determined by Hoechst 33342. Data was normalised to untreated cells and three independent experiments were performed. Mean values ± SEM are shown.

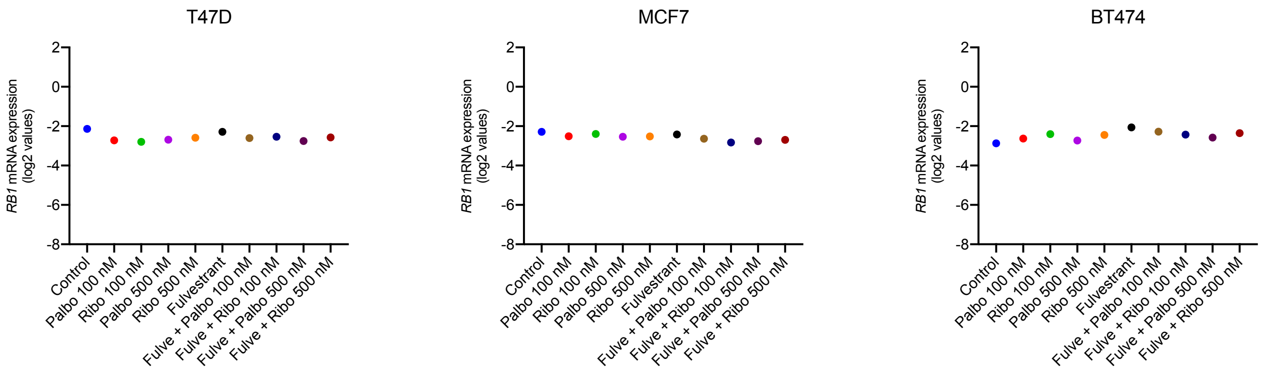

Supplementary Figure 3. *RB1* mRNA expression in breast cancer cell lines after treatment with CDK4/6 inhibitors. T47D, MCF7, and BT474 cells were treated with palbociclib or ribociclib (100/500 nM) with or without fulvestrant for 72 hours and *RB1* mRNA expression was assessed using the nCounter platform.

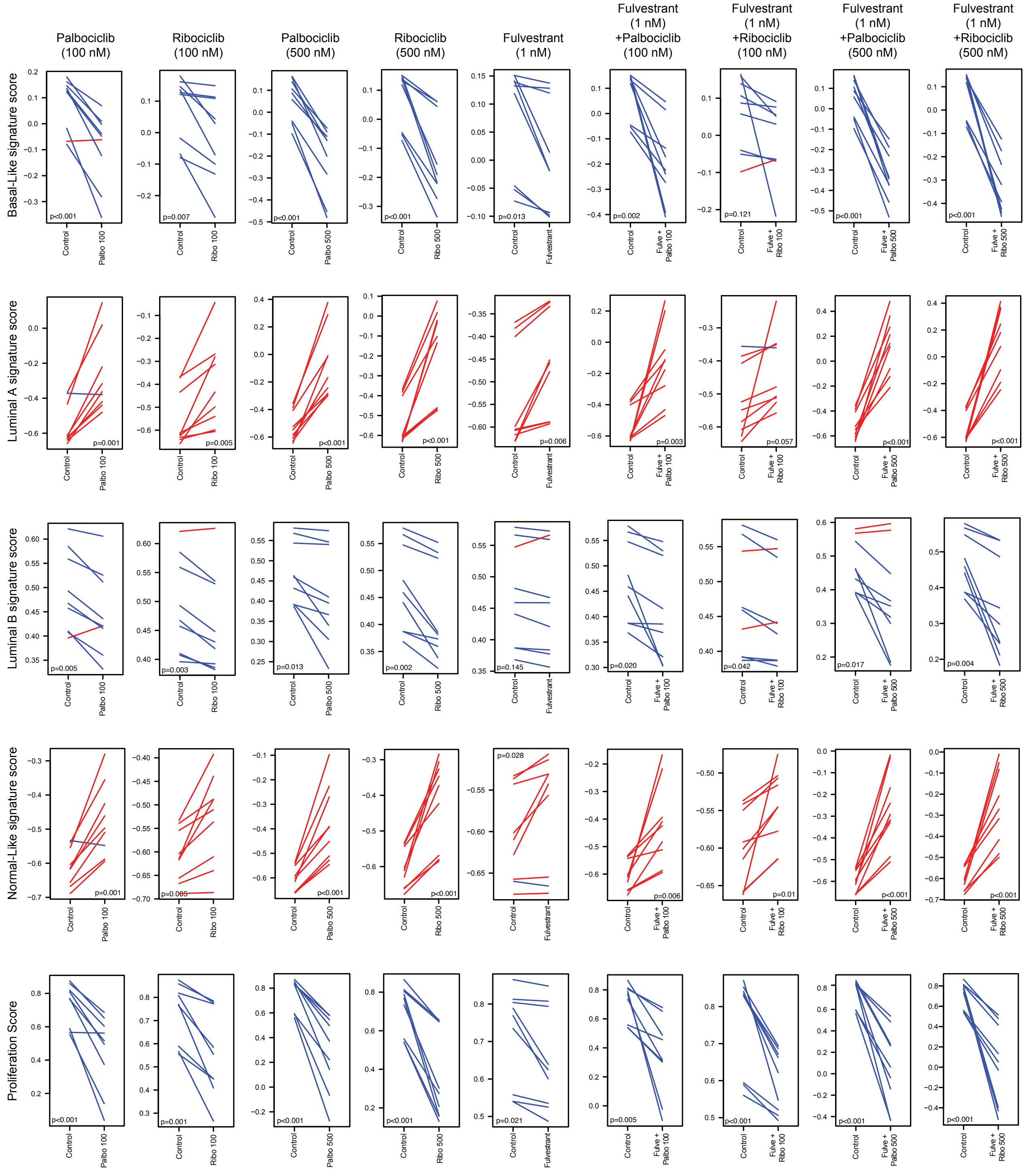

Supplementary Figure 4. Changes in the PAM50 signatures upon treatment with CDK4/6 inhibitors +/- fulvestrant in vitro. Paired samples t-test analyses showing changes in the Basal-Like, Luminal A, Luminal B and Normal-like signatures as well as the proliferation score following treatment with CDK4/6 inhibitors (100 or 500 nM) +/- fulvestrant (1 nM). Three independent mRNA extractions and gene expression analyses were performed for each cell line.

Supplementary Table 1. Differentially expressed genes after treatments. Paired SAM analysis between samples treated with palbociclib 500 nM (negative Score(d)) vs ribociclib 500 nM (positive Score(d)) and palbociclib 500 nM + fulvestrant 1 nM (negative Score(d)) vs ribociclib 500 nM + fulvestrant 1 nM (positive Score(d)) to show differentially expressed genes after treatment (FDR<5%).

Palbociclib 500 nM vs Ribociclib 500 nM			
Gene ID	Score(d)	q-value(%)	PAM50 subtype
KIF2C	4.3014	0	Basal-like
CEP55	4.1995	0	Basal-like
MKI67	4.0133	0	Proliferation score
FOXA1	3.6911	0	Luminal
MYBL2	3.6539	0	Basal-like
KNTC2	3.5728	0	Proliferation score
MDM2	3.5258	0	Normal-like
CXXC5	3.5096	0	Luminal
ESR1	3.2821	0	Luminal
ERBB3	2.8874	0	NA
MELK	2.4888	0	Basal-like
EXO1	2.3875	0	Basal-like
GPR160	1.5991	4.6677	HER2-enriched
CDC20	1.5942	4.6677	Proliferation score
ERBB2	1.4374	4.6677	HER2-enriched
KRT14	1.3944	4.6677	Normal-like
CXCL8	-4.9209	0	NA
KRT5	-3.7660	0	Normal-like
MMP11	-3.6778	0	Luminal
KRT17	-3.6773	0	Normal-like
FGFR4	-3.6420	0	HER2-enriched
MIA	-3.6215	0	Normal-like
ERBB4	-3.5005	0	NA
FOXC1	-3.4982	0	Basal-like
EGFR	-3.0385	0	Basal-like
MLPH	-2.8741	0	Luminal
MAPT	-2.5946	0	Luminal
PGR	-2.3712	0	Luminal
TMEM45B	-2.0383	0	HER2-enriched

Fulvestrant + Palbociclib 500 nM vs Fulvestrant + Ribociclib 500 nM			
Gene ID	Score(d)	q-value(%)	PAM50 subtype
CEP55	3.8310	0	Basal-like
FOXA1	3.6475	0	Luminal
KIF2C	3.4859	0	Basal-like
MDM2	3.3436	0	Normal-like
CXXC5	3.2832	0	Luminal
ERBB3	3.0693	0	NA
ESR1	2.6292	0	Luminal
GRB7	2.2987	0	HER2-enriched
MKI67	2.2081	0	Proliferation score
KNTC2	2.1457	0	Proliferation score
ERBB2	2.0022	0	HER2-enriched
GPR160	1.6941	3.7789	HER2-enriched
ERBB4	-4.4375	0	NA
CXCL8	-4.2148	0	NA
KRT5	-4.1695	0	Normal-like
MIA	-3.9053	0	Normal-like
PGR	-3.7338	0	Luminal
FOXC1	-3.7221	0	Basal-like
MMP11	-3.5001	0	Luminal
FGFR4	-3.4890	0	HER2-enriched
KRT17	-3.0806	0	Normal-like
CD8A	-2.8615	0	NA
MAPT	-2.8144	0	Luminal
EGFR	-2.6807	0	Basal-like
MLPH	-2.2781	0	Luminal
SLC39A6	-2.0990	0	Luminal
ORC6L	-1.6925	2.0294	Basal-like
BCL2	-1.6640	2.0294	Normal-like
CCND1	-1.6226	2.0294	NA

T47D

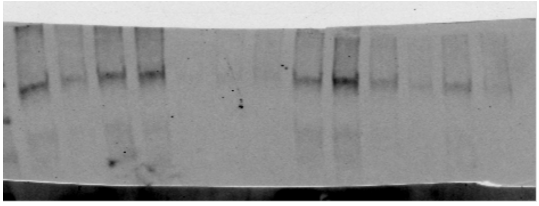

p-Rb

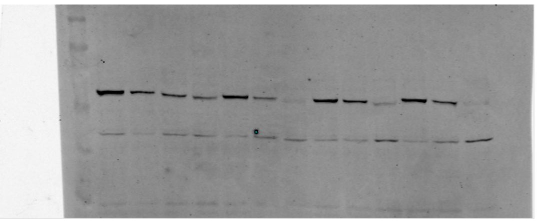

Lamin-B1

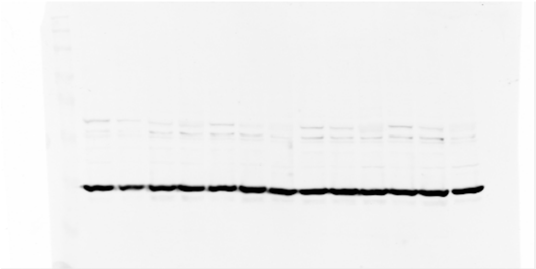

Actin

MCF7

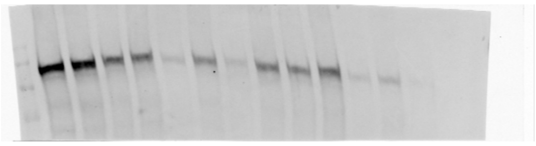

p-Rb

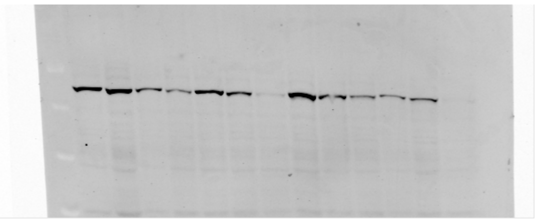

Lamin-B1

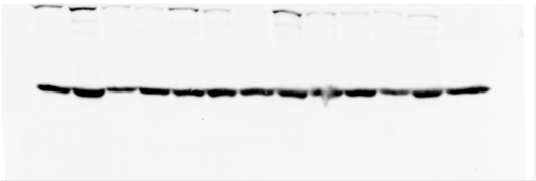

Actin

BT474

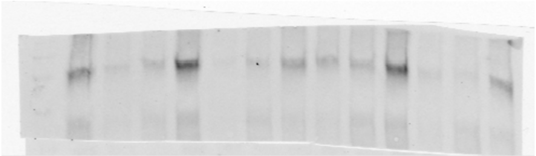

p-Rb

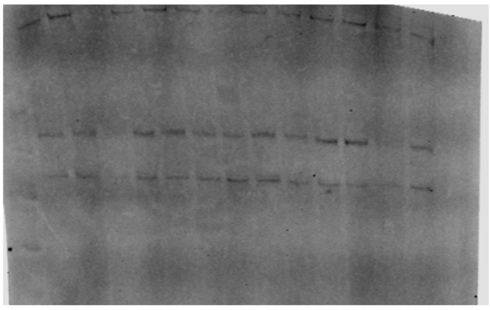

Lamin-B1

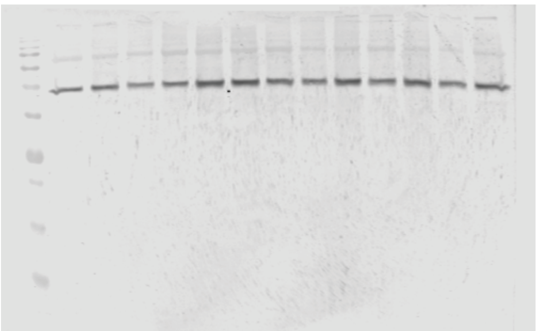

Actin

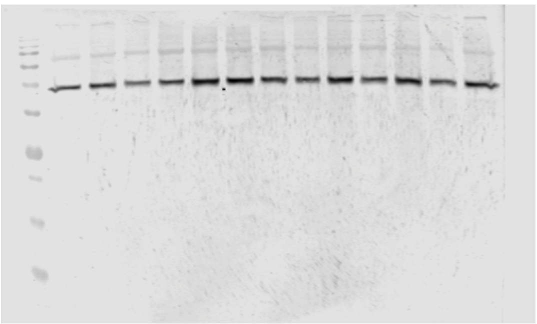

Actin
